# Supplementary material for: Pot-pollen DNA barcoding as a tool to determine the diversity of plant species visited by Ecuadorian stingless bees
Source: PLoS One. 2025 May 14;20(5):e0323306. doi: 10.1371/journal.pone.0323306 (PMC12077722; doi:10.1371/journal.pone.0323306)
Supplement: S1 Table — The species were identified using two distinct methods: molecular biology and morphometric analysis. These methods were developed by two undergraduate students, Esteban Palacios and Ransey Pachacama in 2021 (unpublished information). ID code meaning: H meliponary or nest set, N nest, P pollen sample. (DOCX) [file pone.0323306.s001.docx]

**S1 Table.** Detailed list of Stingless bee species per sampled nest (n=32)

| # sample | Province | Stingless bee species | ID code |
| --- | --- | --- | --- |
| 1 | Orellana | *Melipona eburnea* | H1N4P3 |
| 2 |  | *Melipona scutellaris* | H4N6P2 |
| 3 |  | Unidentified | H4N4P3 |
| 4 |  | *Melipona* sp. | H3N2P1 |
| 5 |  | Unidentified | H4N4P5 |
| 6 |  | *Melipona scutellaris* | H4N5P5 |
| 7 |  | Unidentified | H4N2P4 |
| 8 |  | Unidentified | H4N3P1 |
| 9 |  | *Melipona* sp. | H3N2P2 |
| 12 |  | *Melipona* sp. | H3N1P1 |
| 13 |  | Unidentified | H4N1P3 |
| 14 |  | *Melipona scutellaris* | H4N5P2 |
| 15 |  | *Melipona scutellaris* | H4N5P3 |
| 16 |  | *Melipona scutellaris* | H4N6P3 |
| 17 |  | Unidentified | H4N3P4 |
| 18 |  | *Melipona* sp. | H3N2P3 |
| 19 |  | Unidentified | H4N4P1 |
| 20 |  | Unidentified | H4N2P3 |
| 21 |  | *Melipona eburnea* | H1N4P2 |
| 22 | Loja | Unidentified | H8N2P1 |
| 23 |  | Unidentified | H13N2P1 |
| 24 |  | *Melipona scutellaris* | H8N1P1 |
| 25 |  | *Melipona scutellaris* | H2N7P2 |
| 26 |  | Unidentified | H7N2P |
| 28 |  | Unidentified | H16N2P1 |
| 29 |  | *Scaptotrigona pectoralis* | H10N1P1 |
| 30 |  | *Paratrigona* sp. | H20N2P |
| 31 |  | *Scaptotrigona pectoralis* | H12N2P1 |
| 32 |  | Unidentified | H9N1P1 |
| 33 |  | *Melipona scutellaris* | H2N7P3 |
| 34 |  | Unidentified | H5N1P2 |

The species were identified using two distinct methods: molecular biology and morphometric analysis. These methods were developed by two undergraduate students, Esteban Palacios and Ransey Pachacama in 2021 (unpublished information). ID code meaning: *H* meliponary or nest set, *N* nest, *P* pollen sample
