# Supplementary material for: Correction: Pot-pollen DNA barcoding as a tool to determine the diversity of plant species visited by Ecuadorian stingless bees
Source: PLoS One. 2025 Sep 30;20(9):e0333633. doi: 10.1371/journal.pone.0333633 (PMC12483194; doi:10.1371/journal.pone.0333633)
Supplement: S1 File — (PDF) [file pone.0333633.s001.pdf]

PLOS One

RESEARCH ARTICLE

Pot-pollen DNA barcoding as a tool to determine the diversity of plant species visited by Ecuadorian stingless bees

Joseline Sofía Ocaña-Cabrera[1], Sarah Martin-Solano[2], Jorge Ron-Román[3], Jose Rivas[4], Mutien-Marie Garigliany[4], Claude Saegerman[1]*

1 Research Unit of Epidemiology and Risk analysis applied to Veterinary sciences (UREAR-ULiège), Fundamental and Applied Research for Animal and Health (FARAH) Center, Faculty of Veterinary Medicine, University of Liège, Liège (Sart-Tilman), Belgium, **2** Departamento de Ciencias de la Vida y de la Agricultura, Grupo de Investigación en Sanidad Animal y Humana (GISAH), Carrera de Ingeniería en Biotecnología, Universidad de las Fuerzas Armadas ESPE, Sangolquí, Ecuador, **3** Departamento de Ciencias de la Vida y de la Agricultura, Grupo de Investigación en Sanidad Animal y Humana (GISAH), Carrera Agropecuaria, Universidad de las Fuerzas Armadas ESPE, Campus Politécnico Hacienda el Prado Selva Alegre, Sangolquí, Ecuador, **4** Department of Pathology, Fundamental and Applied Research for Animals & Health (FARAH), Faculty of Veterinary Medicine, University of Liège, Liège (Sart-Tilman), Belgium

* claude.saegerman@uliege.be

Abstract

Identifying the main species of plants from where Ecuadorian stingless bees collect pollen is one of the key objectives of management and conservation improvement for these insects. This study aims to determine the botanical origin of pot-pollen using two barcodes, comparing two methodologies (DNA barcoding versus electron microscopy and morphometric tools) and determine the genus and species of pollen source plants of the main honey-producing stingless bees in Ecuador. As main results, *Prockia crucis*, *Coffea canephora*, *Miconia nervosa*, *Miconia notabilis, Laurus nobilis*, *Cecropia ficifolia*, *Theobroma* sp., *Artocarpus* sp., *Croton* sp., *Euphorbia* sp., *Mikania* sp., and *Ophryosporus* sp., were the genera and species with the highest presence in the nests (n = 35) of three genera of stingless bees of two provinces located in different climatic regions inside the continental Ecuador. Plant species richness in both areas was statistically similar (p-value = 0.21). We concluded that floral sources' molecular identification with the ITS2 region had a higher number of genera and species detected, than the rbcL gene and microscopy tools, for the Ecuadorian landscapes. We confirmed that the foraging behavior of *Melipona* sp., *Scaptotrigona* sp., and *Tetragonisca* sp., could include non-native flora (27%, 12/44 identifications) that provide a rich source of pollen. Stingless beekeepers could use this information to create flower calendars and establish a schedule for better management of stingless bees in secondary and modified environments.

OPEN ACCESS

Citation: Ocaña-Cabrera JS, Martin-Solano S, Ron-Román J, Rivas J, Garigliany M-M, Saegerman C (2025) Pot-pollen DNA barcoding as a tool to determine the diversity of plant species visited by Ecuadorian stingless bees. PLoS One 20(5): e0323306. https://doi.org/10.1371/journal.pone.0323306

Editor: Branislav T. Šiler,Institute for Biological Research, University of Belgrade, SERBIA

Received: August 5, 2024

Accepted: April 4, 2025

Published: May 14, 2025

Copyright: © 2025 Ocaña-Cabrera et al. This is an open access article distributed under the terms of the Creative Commons Attribution License, which permits unrestricted use, distribution, and reproduction in any medium, provided the original author and source are credited.

Data availability statement: All relevant data are within the manuscript and its Supporting Information files.

Funding: The first author was supported by a mobility grant funded by the Académie de

recherche et d'enseignement supérieur (ARES). The funders had no role in study design, data collection and analysis, decision to publish, or preparation of the manuscript.

Competing interests: The authors have declared that no competing interests exist.

Introduction

Pollination services are closely linked to ecosystem stability and biodiversity conservation [1]. Pollinators play a pivotal role in the successful reproduction of flowering plants, thereby promoting genetic diversity and resilience within plant populations [2,3]. Moreover, they ensure the production of seeds and fruits that are vital for human nutrition and food security [4]. The pollination of angiosperms has been found to be beneficial to the health of wildlife, thereby supporting entire food chains (consisting of herbivores, predators, and decomposers) while providing shelter [5]. The fertilisation of plant life by pollinators has been demonstrated to enhance the resilience of these organisms, a factor which is crucial in the context of adapting to environmental changes. Plant diversity helps to sequester carbon, reducing atmospheric CO_2 (carbon dioxide) and aiding in climate change mitigation [6]. The diversity of plant life is also known to stabilise soil, prevent erosion, and regulate water cycles [7].

Since the late 20th century, there has been an ongoing decline in pollinators, a phenomenon that has serious consequences for biodiversity, food security and ecosystem stability [8]. The percentage of pollinating insects, including bees and butterflies, at risk of extinction approached 40% [9]. The decline of bees was attributed to a combination of factors, including habitat destruction, climate change, pesticides, and disease [10–12]. This loss has been recognised as a global crisis by scientists, governments and international organisations. In light of the critical status of insect pollinators, several initiatives have been established on a global scale to ensure their conservation. These initiatives include the International Pollinator Initiative (IPI), the Global Action on Pollination Services for Sustainable Agriculture (FAO), and the Coalition of the Willing on Pollinators. The primary objective of these initiatives is to protect pollinator populations by promoting conservation strategies that integrate agricultural policies and best practices, while also enhancing public awareness [13,14]. The ongoing decline in plant-pollinator interactions is a consequence of the ongoing decline in species of pollinators [15]. The study of these interactions is crucial for preventing the loss of biodiversity in plant communities, as many species are dependent on specific pollinators. A drop in pollination network vitality could limit food sources for other wildlife, affecting their well-being and potentially impacting human food security. A comprehensive understanding of these interactions can help reduce economic losses from pollination services and industries that depend on effective pollination [2,16].

The floral richness of the tropics is particularly affected by the decline of pollinator populations, as the maintenance of their biodiversity is highly dependent on native pollinators, and these are the invertebrates least able to adapt rapidly to changing climates and thus most vulnerable to extinction [17,18]. The Amazon rainforest is characterized by its biodiversity and ecological significance. It plays a crucial role in regulating the climate, storing carbon, and providing essential ecosystem goods and services. These include oxygen, fresh water, medicinal and economic benefits for indigenous communities, to the existence of human life, and future generations [19]. Animals pollinate around 94% of native tropical plants [20], and in the Amazon rainforest, 54% of these animal pollinators are bees [21].

Stingless bees are the main pollinators inside tropical ecosystems, moving from flower to flower until they find the most suitable food [22–24]. Daily-ranging patterns of stingless bees depend on each species' foraging behaviour, which may differ in space use, detection, and foraging distance. For example, the Asian stingless bee (*Tetragonula biroi*) has a short flight range, 250–500 meters (m), as do Australian stingless bees (*T. carbonaria*) have (333–712 m) [25], while American stingless bee genera (*Melipona* sp. and *Trigona* sp.) have longer flight ranges of 1.5 and 2.1 km respectively [26]. The role of native fauna in stabilizing ecosystem services is to buffer the effects of climate change [27]. However, there is evidence of high thermal thresholds that may mark weak selection processes or strong evolutionary constraints [28]. An abnormal accumulation of polyols (mannitol and sorbitol), which act as preventive molecules against protein denaturation or cell inactivation, has been found in insects [29,30]. To mitigate the effect of all these changes it is necessary to understand the basic natural functioning of bees and their relationship, especially with floral sources, directly concerned with the upkeep of crops for human consumption [31].

Foraging of floral sources by stingless bees does not follow a described pattern. In general, it has been concluded that foraging occurs according to the need of the nest and the availability of sources [32], i.e., temporal specialization intervals [33,34].

To obtain a better understanding of the pollen sources of bees, different techniques have been developed, such as light microscopy and pattern recognition carried out by a visual expert [35], phase contrast and dark field microscopy [36], and vibrational spectroscopy [37,38]. Moreover, even software and artificial intelligence development now allow for automated pollen recognition [39]. Due to continuous scientific improvements, molecular biology techniques have been included in pollen research [40,41]. Indeed, barcoding is a technique that allows species recognition through the characterization of standard genes [42]. In the case of land plant barcoding, selected DNA regions must satisfy the following criteria: (i) be routinely amplifiable; (ii) to have a sufficient variation to differentiate closely related species and yet also show sufficient sequence consistency to ensure that intraspecific variation does not confound species assignation; (iii) to have specific amplification unsusceptible to the amplification of other DNA regions [43]. An effective DNA barcode region possesses conserved flanking sites for developing universal PCR primers for wide taxonomic applications. Nuclear regions, such as the Internal transcribed spacer 2 (ITS2) provide more information than barcoding based on the organellar gene [44,45]. Nuclear DNA (nDNA) exhibits faster rates of evolution in comparison to chloroplast DNA (cpDNA), resulting in the accumulation of a greater number of mutations over time. The genetic variation that results from this process facilitates the distinction between closely related species [46,47]. Multiple gene copies from nuclear genome increase the species resolution. The biparental inheritance in angiosperms capture the recombination and hybridization, thereby contributing to the augmentation of diversity identification [48–50].

The ribulose 1,5-biphosphate carboxylase oxygenase (rbcL) gene is a constituent of chloroplast DNA and represents a valuable marker due to its documented ease of amplification when using primers that apply to all land plants. Additionally, it has been demonstrated that this gene is capable of identifying taxa at the genus and family levels, and has also been shown to be an effective species-level identifier in comparative data mining tests. Furthermore, it is the most extensively characterized plastid coding region in GenBank [51,52]. The low mutation rate of this gene, when used in conjunction with other markers, enhances analysis at both ecological and evolutionary levels [53]. Some organellar genomes, like those in organelles like mitochondria and chloroplasts, change very slowly. This can make closely related but distinct species appear genetically identical. This can mask true species diversity and lead to an underestimation of richness [54,55]. Conversely, an overestimation may occur due to species interbreeding. In such instances, organellar DNA from one species may be retained in the hybrid, while nuclear DNA remains distinct. This phenomenon can result in the erroneous classification of hybrids as new species. Nevertheless, a combination of nuclear and chloroplast markers is frequently employed for the purpose of robust species identification [56,57]. The ITS2 region and the rbcL gene were selected as markers due to their high universality, sequence variability, and ease of amplification. The combination of these markers is further enhanced by the following factors, the ITS2 region has been shown to provide high resolution at the species level, but can

be difficult to amplify in some plant groups. In contrast, the rbcL gene is easy to sequence and works in all land plants, but has lower species discrimination power. The utilization of these two markers serves to mitigate the occurrence of erroneous identifications and taxonomic misclassifications [58–60].

South America has a low level of scientific data production with metagenomics tools and microbiome studies [61]. Ecuador is not an active player in biodata generation often due to the technology gap, but its unique biodiversity has great potential to contribute to global projects related to this field [62], especially for the knowledge of Ecuadorian biodiversity. To contribute to the knowledge of biodiversity through the identification of plants using pollen collected by stingless bees in two provinces of Ecuador, this study aims to (i) determine the taxonomy of plants using pot-pollen from stingless bee nests, using two the ITS2 region and rbcL gene for barcoding, to (ii) make a comparison between the scope of taxonomic identification obtained by microscopy and by DNA, and to (iii) evidence the difference of the main pollen sources for stingless bee genera in two environmentally different areas of Ecuador (Fig 1).

Materials and methods

Ethical aspects

The applicable legislation was applied during the manipulation of specimens and habitat involved in this study. Habitat was not disturbed or altered in any significant way, and no animals were harmed in the process of obtaining the samples.

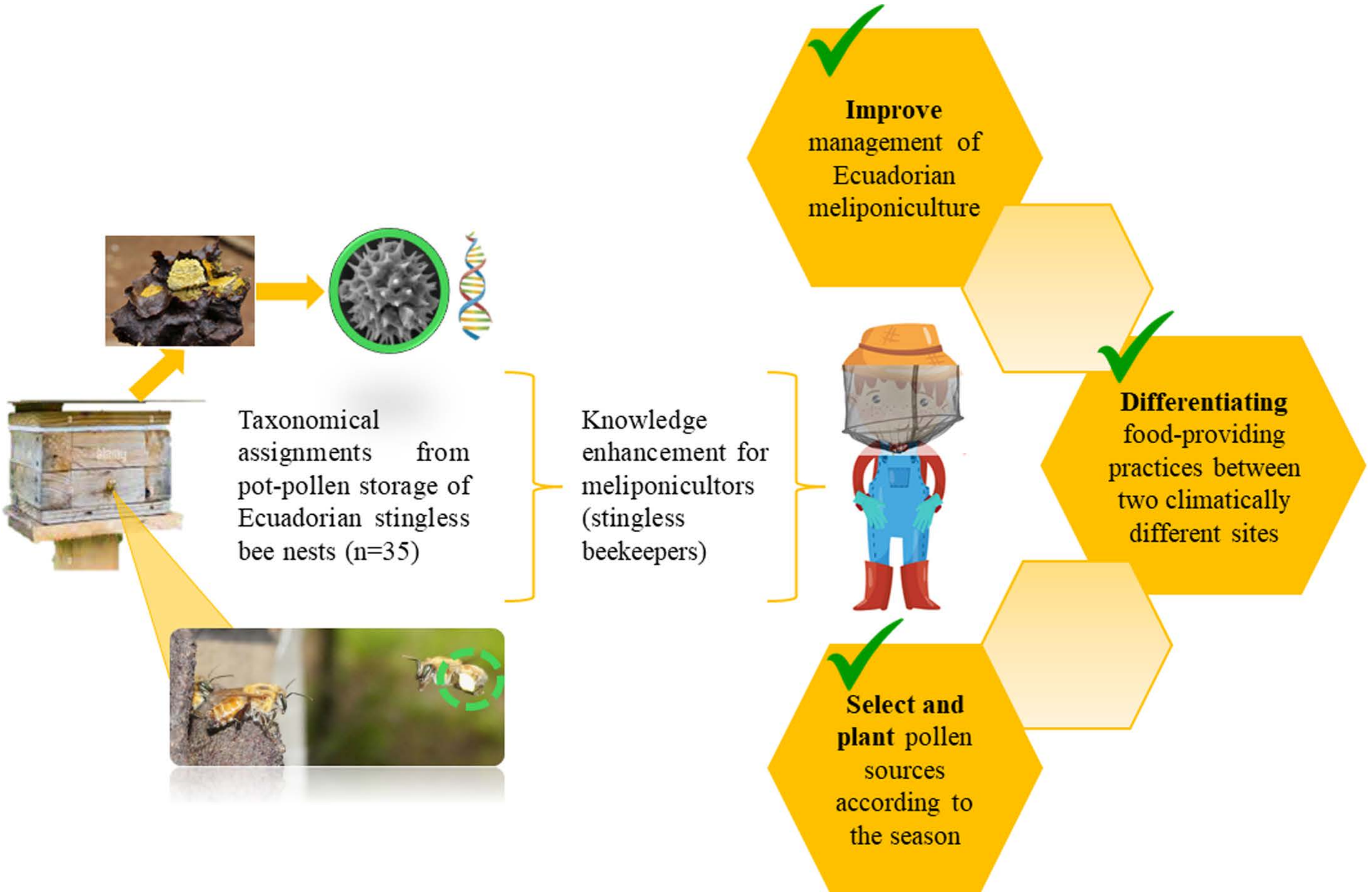

Fig 1. Graphical abstract of the study.

https://doi.org/10.1371/journal.pone.0323306.g001

Pollen as a product of a stingless bee nest is classified as a food supplement and/or natural medicinal product. The objective of the collection was explained to each owner, and field site access was obtained by each meliponicultor (from Orellana and Loja province) under the terms of informed consent as part of the Synergy Project, which was approved under the number CVGP-0025–2017 by the Universidad de las Fuerzas Armadas ESPE, Ecuador. Meliponicultors signed a consent for the collection of pollen samples from their stingless bees' nests in August-September, December 2018, and March 2019.

Study areas

The sampling areas were the Amazon rainforest (Orellana province) and the southern highland region with dry tropical forests (Loja province) (Table 1). In Ecuador, the summer lasts from September to February. For Orellana province, the average temperatures and precipitations are 25 °C and 127 mm, respectively. The average temperature and precipitation in Loja province are 21 °C and 36 mm.

Pollen sampling

This cross-sectional survey randomly selected 35 pot-pollen samples from technical stingless bee nests (**S1 Table**) belonging to 4 meliponaries in Orellana and 11 meliponaries in Loja, with several samples from the same nest set (n = 21 and n = 14, respectively).

We collected pollen samples only from sealed pots of nests that were sampled once during the four months.

Pollen wall lysis and DNA isolation

Fifty milligrams of pollen were weighed into a 2 mL Eppendorf tube. 500 uL of buffer lysis of Macherey-Nagel NucleoSpin Food kit (Macherey-Nagel, Düren, North Rhine-Westphalia, Germany) and 500 uL of ceramic beads (1mm) were added. We vortexing samples to achieve a homogeneous mixture. Each tube was placed into a Tissuelyser II instrument (QIAGEN®) for 3 min, 30 Hz. The tubes were centrifuged for 2 min at 5000 g. 10 uL of proteinase K (20 mg/mL) was added and incubated for 30 min, 65 °C.

Total genomic DNA was extracted using the Macherey-Nagel NucleoSpin® Food kit (Macherey-Nagel, Bethlehem, Pennsylvania, USA), following the "Isolation of genomic DNA from honey or pollen" supplementary protocol. Negative control was included in the experiment, consisting of sterilized water instead of pollen. Finally, the DNA purity of each sample was measured using NanoDrop ® Spectrophotometer ND-1000, ISOGEN Life Science.

Real-time PCR

Temperature and time conditions [hold stage 95 °C 180 sec (95 °C 30 sec, 60 °C 30 sec, 72 °C 45 sec) x 40 cycles, melting stage 95 °C 15 sec] were established for the amplification of both regions (Table 2). The amplification of each region was carried out separately and in duplicate per sample (Table 3) using Luna® Universal Probe One-Step RT-qPCR Kit.

Table 1. Geographical localization of sampling zones.

Province	Locality	Geographical localization
Orellana	Dayuma	0°40'16"S, 76°52'54"W
Loja	Celica	0°40'10"S, 80°04'90"W
	Pindal	0°30'57"S, 79°59'04"W
	Puyango	0°30'57"S, 79°58'27"W

S: south. W: west

https://doi.org/10.1371/journal.pone.0323306.t001

Commercial pollen from Belgium was used as a positive control and sterile water as a negative control. The PCR assembly was conducted in two chambers: one for the preparation of the master mix and one for the addition of the DNA. We used this endpoint PCR modality to assess the quality of pollen DNA through C_T (cycle threshold) values.

Illumina sequencing

Amplicon libraries were prepared according to the Illumina 16s metagenomic workflow protocol [66], with adaptations. PCR1 was done for both amplicons separately (ITS/rbcL) and then mixed before cleaning up with Ampure beads (cf 16s-metagenomic-library-prep-guide-15044223-b, page 8). For this PCR1, we used 40 cycles instead of 25, and Q5® High-Fidelity DNA Polymerase (M0491), but PCR2 was processed with Kapa HiFi polymerase like in the 16s Illumina protocol. PCR2 was done with 5 µl of purified PCR1 product. At the end of PCR2, all libraries were dually indexed. Different combinations of indexes (Nextera Index Kit - Index 1 (i7) Adapters, from N708 to N712 and Index 2 (i5) Adapters, from N501 to N508. Oligonucleotide sequences © 2015 Illumina, Inc. All rights reserved) were used for each sample.

PCR2 products were then purified with AMpure beads (cf 16s-metagenomic-library-prep-guide-15044223-b, page 13), and amplicon QC was done on QIAxel (size profile) (QIAGEN®, Germany).

PCR2 products were quantified and normalized at 7 ng/µl using Quant-iT™ PicoGreen™ dsDNA Assay Kit (ThermoFisher Scientific). We generated an equimolar pool at 5 ng/µl. Before proceeding to Illumina MiSeq paired-end 300 bp, the final pool was quantified by qPCR using KAPA SYBR® FAST qPCR Kits (Sopachem) with Library Quantification DNA Standards Illumina from Roche. 8.5 PM of the denatured final pool was loaded on a Miseq 600 cy v3 kit. As a control step, we added 10%pf Phix (PhiX Control v3), a ready-to-use control library for Illumina sequencing runs.

Bioinformatic analysis

The process started with 2 213,285 forward sequences and the same number of reverse sequences for ITS2 and rbcL. We adapted steps 1–3 of Quantitative Insights Into Microbial Ecology (QIIME 2) workflow for metabarcoding analysis

Table 2. Primers sequence information.

Gen	Sequence (5’ ◊ 3’)	Reference
rbcLaF (forward)	ATGTCACCACAAACAGAGACTAAAGC	[63]
rbclr506 (reverse)	AGGGGACGACCATACTTGTTCA	[64]
ITS-3p62plF1 (forward)	ACBTRGTGTGAATTGCAGRATC	[65]
ITS-4unR1 (reverse)	TCCTCCGCTTATTKATATGC	

Note: for ITS2 uncommon letter the interpretation is: *B is C, T or G, R is A or G, K is G or T.*

https://doi.org/10.1371/journal.pone.0323306.t002

Table 3. PCR preparation by microtube.

Product	Quantity
Luna ® Universal Probe qPCR Master Mix	10 µL
Nuclease-free water	6 µL
Primer forward (ITS2 or rbcL)	2 µL
Primer reverse (ITS2 or rbcL)	2 µL
Sample DNA	5 µL
Total volume	**25 µL**

µL: microlitres

https://doi.org/10.1371/journal.pone.0323306.t003

(18S/16S rRNA) with already-demultiplexed fastq files (https://github.com/BikLab/BITMaB2-Tutorials/blob/master/QIIME2-metabarcoding-tutorial-already-demultiplexed-fastqs.md), to specific information of ITS2 and rbcL reads. To illustrate, we avoided the demultiplexed command in step 1 "Importing data, summarize the results, and examining quality of the reads". The values for truncated bases of sequences in step 2 "Quality controlling sequences and building Feature Table and Feature Data" were modified. The DADA2 plugin in Qiime 2 employs a default filtering process that excludes any PhiX reads from the sequencing data and filters out chimeric sequences. A quality plot was consulted to eliminate noise and establish the requisite parameters. It was noted that the mean quality score was 34, along the initial bases, which led to the decision to set --p-trim-left = 0 for both, forward and reverse reads. The quality plot, in turn, informed the decision to set the parameter --p-trunc-len = 250 for forward reads and --p-trunc-len = 200 for reverse reads, sites after which the quality drops significantly. After the last step, four samples were removed. This decision was made due to the limited number of sequences available for each sample, and the substandard quality of the sequences. Due to the sampling site's biodiversity, we dereplicate the sequences in Amplicon Sequence Variants (ASVs). Following the filtration and denoising processes, 541,174 paired-end reads were obtained for ITS2, and 301,612 were received for rbcL.

The DUBOIS curated NCBI_ITS2_Viridiplantae and NCBI_rbcL_Viridiplantae [67] database (both dereplicated-restricted) was employed to assign the taxonomy of ITS2 and rbcL unknown sequences in step 3 "Assigning Taxonomy". The percentage of the similarity threshold for assignment to the species level was 95%. The training classifier was configured using the classify-consensus-blast algorithm, with a max accept value of 1. The final assigned reads were 1,007. However, the reverse rbcL reads demonstrated a consistent lack of quality (step 2), which complicated the pairing and analysis of pairs of reads. For the taxonomic assignment, the focus was exclusively on the rbcL forward reads. However, the classifiers (Blast and vSearch) were unable to identify reliable assignments. Manual mapping against specific reference databases found in NCBI was then performed for rbcL barcode, although this process was more laborious and time-consuming.

Finally, step 4 "Summarizing Feature Table and Feature Data" of the workflow for metabarcoding analysis was followed similarly.

Scanning electron microscopy (SEM) and morphometry method

We used the method developed in our previous work [33], in which high-quality 2D SEM images and morphological anatomical points were used to identify plant families and genera, to compare the results obtained in the present study.

Statistical analysis

Alpha diversity was used to compare samples from the two provinces. To measure the branching length between the taxonomic assignments of the ASVs, Faith's Phylogenetic Diversity (PD) was used. The Alpha diversity calculation used the total number of samples ($n = 35$).

The significance of the difference between biodiversity values was measured using the pairwise Kruskal-Wallis Test.

Results

A total of 35 samples were obtained for inclusion in the study. The ratio absorbance 260/280 of DNA extracted from the samples was 1.64–2.21 ng/μL. The C_T for ITS2 region was 13.696–34.794 (22.24 ± 0.27) while the C_T for rbcL gene was 15.901–39.336 (25.78 ± 0.33).

Following the quality control step in the bioinformatics analysis, samples 9, 10, 27, and 35 were excluded due to the poor quality of the reads generated after sequencing. The total number of taxonomic assignments made for the ITS2 sequences at the family level was 26, at the genus level was 51, and at the species level was 204. In the case of the rbcL sequences, no taxonomic assignments were made at the family level; however, a single assignment was identified at the genus level, and 61 taxonomic assignments were made at the species level. Although 31 samples successfully passed the quality filtration process, obtaining the aforementioned number of taxonomic assignments was only possible from 28

pollen samples (GenBank accession numbers from SAMN46265110 to SAMN46265137). No valid taxonomic assignments were obtained from any DNA barcode for samples 11, 28, and 32.

The taxonomic assignments obtained from the two DNA markers represented families, genera, and species that were repeated. Consequently, a total of 64 ASVs were identified as unique and distinct taxa, of which 4 (6%) were classified at the family level, 34 (53%) at the genus level, and 26 (41%) at the species level.

We identified 58% (37/64) of the ASVs through the ITS2 region, and 42% (27/64) through the rbcL gene. Taxonomy identification scope was superior using pollen DNA analysis than morphology and geometric morphometry (SEM identification) analysis, since it was not possible to reach species with the last methodology (Fig 2).

The SEM methodology enabled the identification of up to five families within the same sample. Using the ITS2 region we were able to identify at least four different families (six species) per sample, whereas using the rbcL gene, it was possible to identify only four families (four species) within the same sample.

Regarding the alpha diversity of the samples based on the phylogenetic distribution (Fig 3), there was no significant difference between species richness in the two sampling sites (p-value = 0.21).

We were able to differentiate the pollen sources according to the seasons, August and September were dry months for both study sites (Amazonian regions and southern highlands), while December and March were rainy months (Fig 4).

During dry months for Orellana province, we found 24 miscellaneous sources, mainly Melastomataceae, *Artocarpus* sp., *Croton* sp., *Euphorbia* sp., *Prockia crucis*, while during a rainy month (December) 19 different pollinic plants were identified, such as *Theobroma* sp., *Prockia crucis*, *Miconia* sp2., Anacardiaceae, *Artocarpus* sp. (Table 4).

In March, the rainiest month for Loja province, we identified 23 plant sources mostly *Cecropia ficifolia*, *Coffea canephora*, *Coffea* sp., *Mikania* sp1., *Ophryosporus* sp., compared to only six in the dry month (September), *Coffea canephora*, *Prockia crucis*, *Miconia nervosa*, *Theobroma* sp., *Laurus nobilis*, and *Cecropia ficifolia* (Table 5).

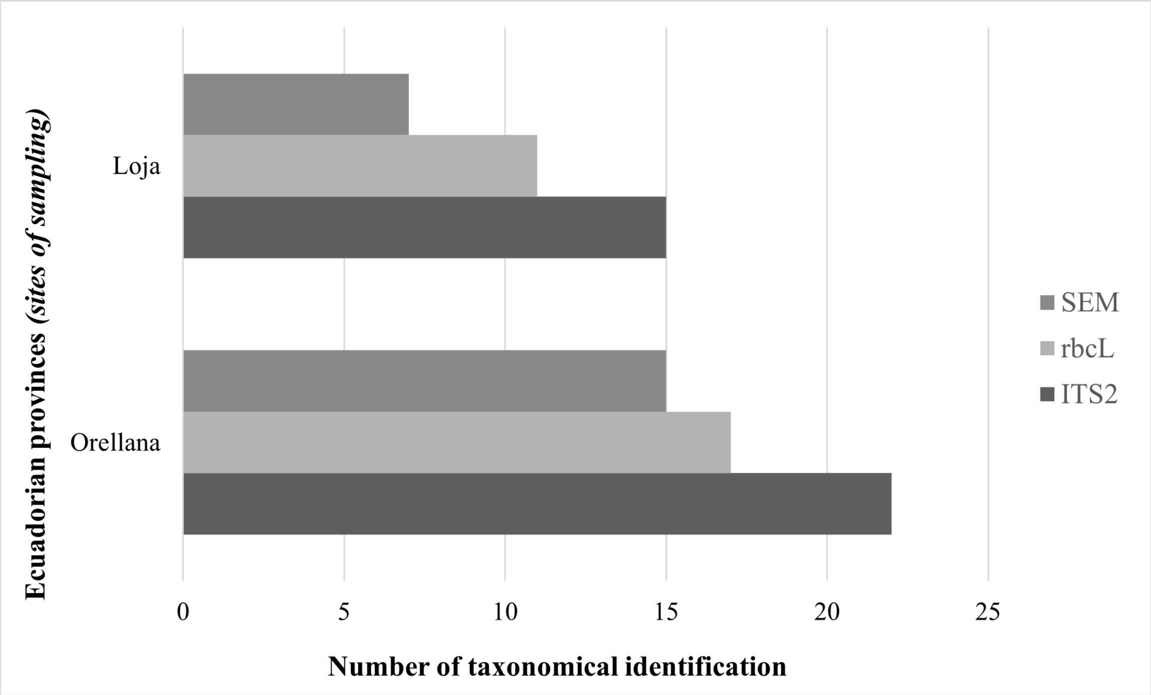

Fig 2. Scope comparison of plants identifications using three different methods: barcode with ITS2 region, barcode with rbcL gene, scanning electron microscopy and morphometry identification (SEM).

https://doi.org/10.1371/journal.pone.0323306.g002

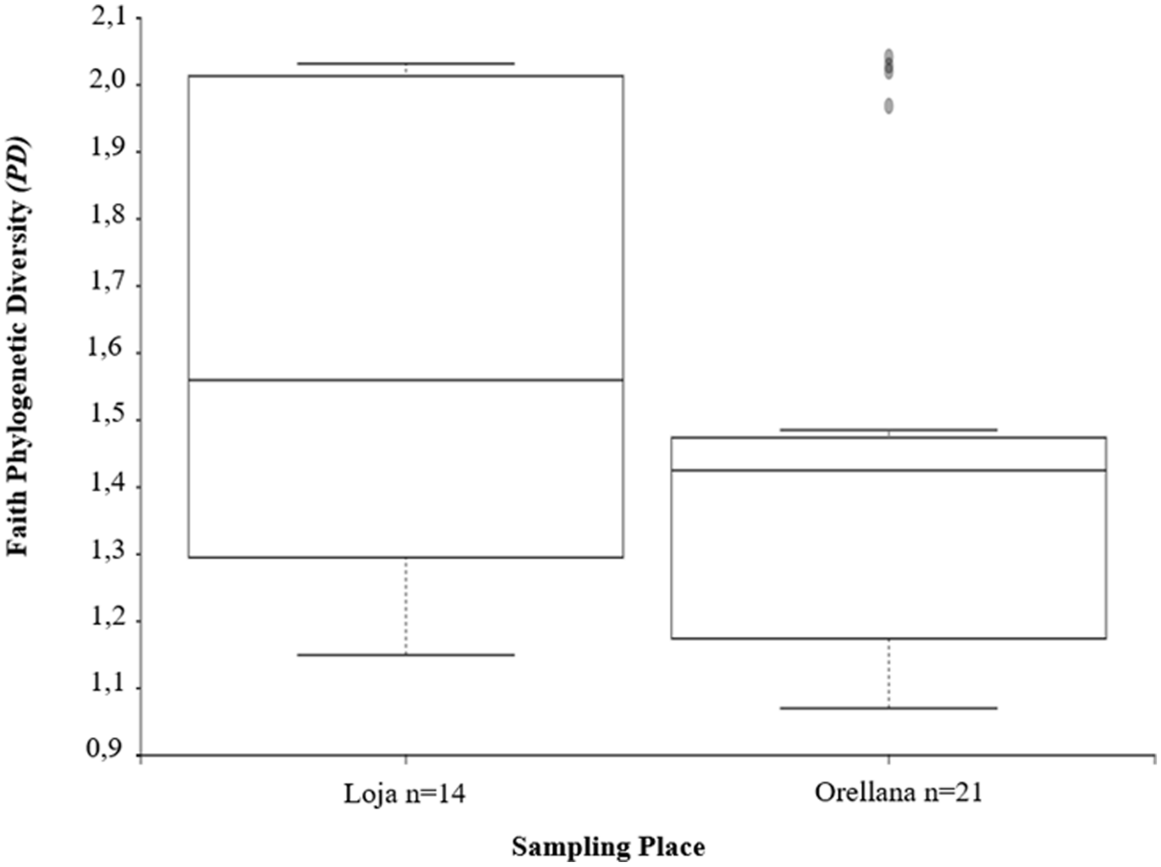

Fig 3. Alpha diversity boxplot by sampling place, tropical dry forest (Loja), Amazonian rainforest (Orellana).

https://doi.org/10.1371/journal.pone.0323306.g003

Once, we identified 64 ASVs, we found that 19% (12/64) of the taxonomic identifications were considered as introduced flora (Table 6) for Ecuadorian territory (Fig 5), with a variety of weeds (33%), shrubs (50%) and trees (17%).

Discussion

We were able to identify 64 plants as pollen sources at different taxonomic levels, 6% at the family level, 41% at the species level, and 53% at the genus level, using DNA barcode analysis. We differentiate the seasonal pollen sources for two climatological distinct regions in continental Ecuador, the southern highland with dry tropical forest (Loja province) and the Amazonian rainforest (Orellana province). For both sites, the main identifications based on the highest abundance (number of reads per ASV) of plants present per sample were *Prockia crucis*, *Coffea canephora*, *Miconia nervosa*, *Laurus nobilis*, *Theobroma* sp., *Miconia notabilis*, *Artocarpus* sp., *Croton* sp., *Euphorbia* sp., *Cecropia ficifolia*, *Mikania* sp., and *Ophryosporus* sp.

The ITS2 region obtained the major scope for taxonomic assignments, because its variability allows for distinguishing closely related species. Additionally, its conserved regions make it valuable for designing universal primers [44]. The internal transcribed spacer 2 length is 180–390 bp for plants [68]. Meanwhile, the rbcL gene has a full length of 1400 bp [69]. RbcL gene is a good region for phylogenetic studies due to a low mutation rate that maintains sequence stability over generations, allowing evolutionary relationships between plants to be mapped. Its highly conserved sequence also enables broad applicability across different taxa [63].

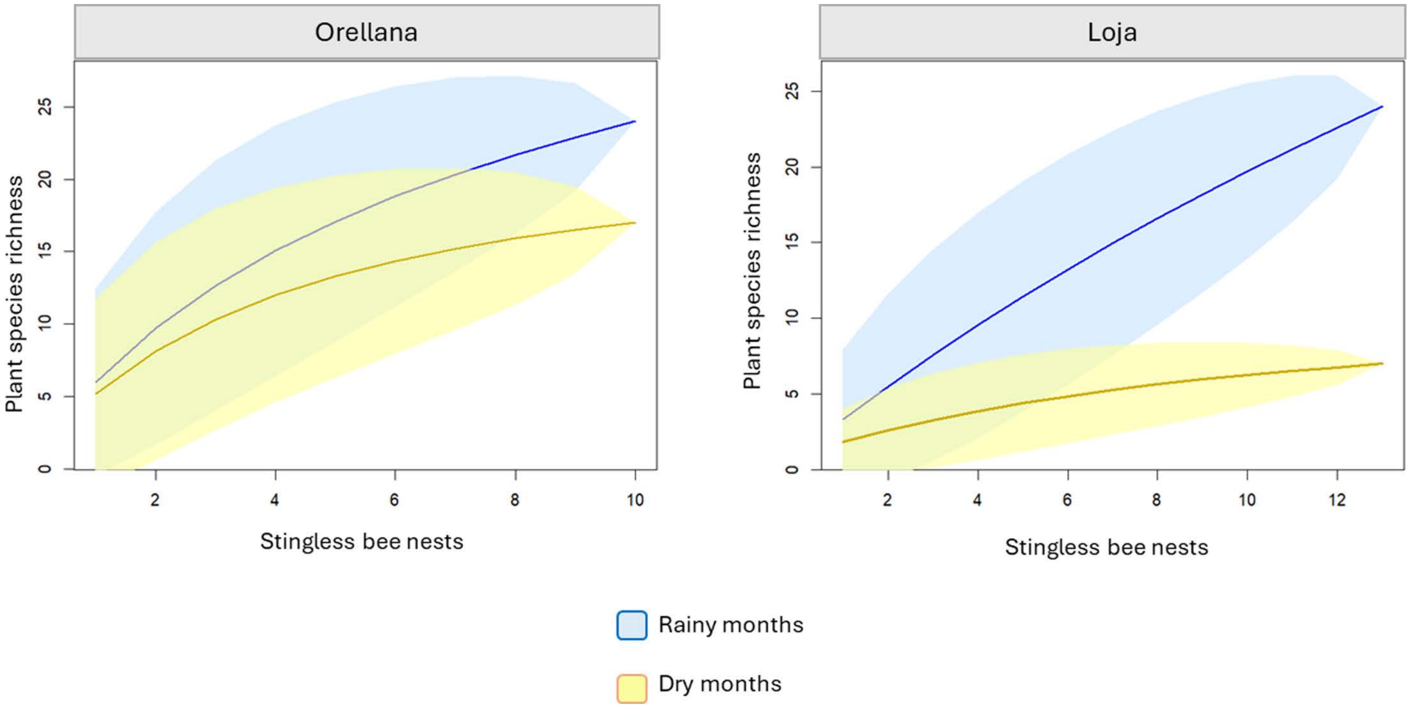

Fig 4. Seasonal acumulation curve (rarefaction) for plant taxonomical assignment according to the stingless bee nests sampled. Rainy months (blue line) December 2018, and March 2019. Dry months (yellow line) August, and September 2018. The 95% of confidence interval (blue and yellow transparency) is also indicated.

https://doi.org/10.1371/journal.pone.0323306.g004

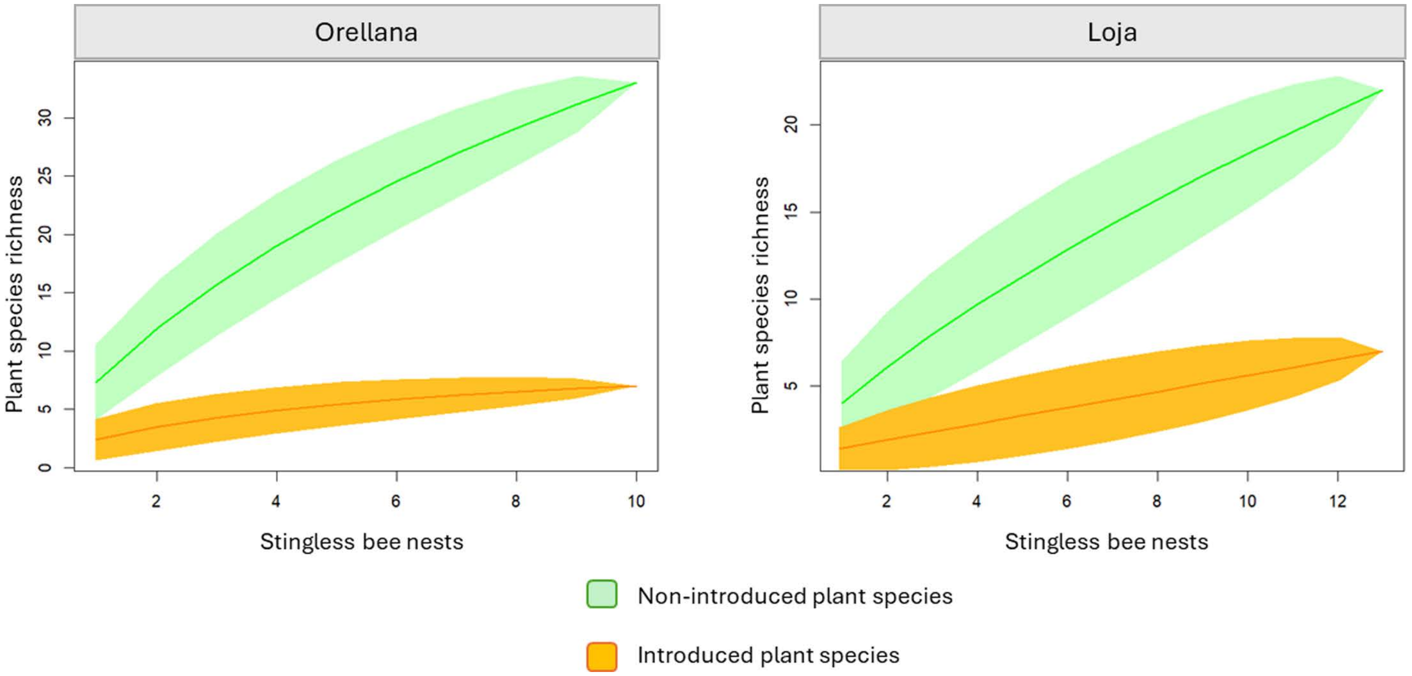

Fig 5. Type of flora acumulation curve (rarefaction) for plant taxonomical assignment according to the stingless bee nests sampled. Non-introduced plants (green line), and introduced plants (orange line) for Ecuador. 95% of confidence interval (green and orange transparency).

https://doi.org/10.1371/journal.pone.0323306.g005

The rbcL barcode as a gene in the plastid DNA began to be less recommended for the analysis of pollen DNA since it is not present in all pollen grains. However, it is essential to maintain it for reliable plant identification with close taxa [70] and the quantitative data that produce at least at the family level. The majority of the families identified in this study fall into this category, including Asteraceae, Brassicaceae, Fabaceae, Moraceae, Rosaceae, and Salicaceae [71]. The elevated number of species-level identifications in this study may appear surprising, but the efficacy of rbcL as a DNA marker for species-level identification has been demonstrated in specific groups of plants that show greater interspecific variation, i.e., greater sequence divergence, which allows species resolution. This feature has been identified in species belonging to the families Asteraceae, Fabaceae, Poaceae, and Orchidaceae [72–75]. However, it is important to recommend the use of the matK gene in conjunction with the rbcL gene to improve species resolution in similar studies.

The combination of ITS2 and rbcL markers was found to facilitate more precise species-level identification [76] than that achievable with either marker in isolation [77–82]. The optimisation of the methods employed was also a factor that enriched our results. Increasing the number of PCR cycles from 25–35 to 40 has a small impact on species-level identification [83]. However, this increase allows the amplification of ITS2 sequences from other plants, which would not be possible with fewer cycles. During our study, the use of 40 cycles for the PCR amplification may have made the ITS2 region the best identification marker. The next-generation sequencing method has been a popular way to analyse pollen [84]. In this study, the MiSeq system worked, as is usual, with 300 pb paired-end reads, which is a key tool in the case of ITS region analysis because this specific length recovers the informative sequence ITS2 and ITS1 [85]. However, MiSeq is a short-read NGS sequencing platform. Consequently, we would recommend HiFi-based platforms such as PacBio, which

Table 4. Seasonal pollen references for Orellana province (amazon region).

		**August – September 2018 (dry months)**		**December 2018 (rainy month)**
	1	Melastomataceae	1	*Theobroma* sp. (Malvaceae)
More	2	*Artocarpus* sp. (Moraceae)	2	*Prockia crucis* (Salicaceae)
↓	3	*Croton sp* (Euphorbiaceae)	3	*Miconia* sp2. (Melastomataceae)
	4	*Euphorbia* sp. (Euphorbiaceae)	4	Anacardiaceae
	5	*Prockia crucis* (Salicaceae)	5	*Artocarpus* sp. (Moraceae)
	6	*Schefflera* sp. (Araliaceae)	6	*Choerospondias axillaris* (Anacardiaceae)
	7	*Miconia notabilis* (Melastomataceae)	7	*Coffea canephora* (Rubiaceae)
	8	*Theobroma* sp. (Malvaceae)	8	Melastomataceae
Less	9	*Dendropanax* sp. (Araliaceae)	9	*Miconia notabilis* (Melastomataceae)
	10	*Bellucia grossularioides* (Melastomataceae)	10	*Schefflera* sp. (Araliaceae)
	11	*Solidago* sp. (Asteraceae)	11	*Eugenia* sp. (Myrtaceae)
	12	*Triolena amazonica (*Melastomataceae)	12	*Baccharis* sp. (Asteraceae)
	13	*Calyptranthes* sp. (Myrtaceae)	13	*Mikania cordifolia* (Asteraceae)
	14	*Miconia affinis* (Melastomataceae)	14	*Brassica napus* (Brassicaceae)
	15	*Psidium* sp1. (Myrtaceae)	15	*Acmella* sp. (Asteraceae)
	16	*Brassica napus* (Brassicaceae)	16	*Erigeron sumatrensis* (Asteraceae)
	17	*Coffea canephora* (Rubiaceae)	17	*Mauria* sp. (Anacardiaceae)
	18	*Trophis caucana* (Moraceae)	18	*Aster* sp. (Asteraceae)
	19	*Miconia tococoidea* (Melastomataceae)	19	*Muntingia calabura* (Muntingiaceae)
	20	*Baccharis* sp. (Asteraceae)		
	21	*Ficus andicola* (Moraceae)		
	22	*Triplaris melaenodendron* (Polygonaceae)		
	23	*Aster* sp. (Asteraceae)		
	24	*Ficus* sp. (Moraceae)		

https://doi.org/10.1371/journal.pone.0323306.t004

Table 5. Seasonal pollen references for Loja province (southern highland region).

		September 2018 (dry month)		March 2019 (rainy month)
	1	*Coffea canephora* (Rubiaceae)	1	*Cecropia ficifolia* (Urticaceae)
More	2	*Prockia crucis* (Salicaceae)	2	*Coffea canephora* (Rubiaceae)
↓	3	*Miconia nervosa* (Melastomataceae)	3	*Coffea* sp. (Rubiaceae)
	4	*Theobroma* sp. (Malvaceae)	4	*Mikania* sp1. (Asteraceae)
	5	*Laurus nobilis* (Lauraceae)	5	*Ophryosporus* sp. (Asteraceae)
	6	*Cecropia ficifolia* (Urticaceae)	6	*Withania* sp. (Solanaceae)
			7	*Leucaena* sp. (Fabaceae)
			8	*Psidium* sp2. (Myrtaceae)
Less			9	*Trophis caucana* (Moraceae)
			10	*Dillenia* sp. (Dilleniaceae)
			11	*Schefflera* sp. (Araliaceae)
			12	*Tapirira guianensis* (Anacardiaceae)
			13	*Theobroma* sp. (Malvaceae)
			14	Myrtaceae
			15	*Swartzia polyphylla* (Fabaceae)
			16	*Brassica napus* (Brassicaceae)
			17	*Secale cereale* (Poaceae)
			18	*Triticum turgidum* (Poaceae)
			19	*Baccharis* sp. (Poaceae)
			20	*Alternanthera* sp. (Amaranthaceae)
			21	*Pisonia* sp. (Nyctaginaceae)
			22	*Ageratina adenophora* (Asteraceae)
			23	*Bougainvillea praecox* (Nyctaginaceae)

https://doi.org/10.1371/journal.pone.0323306.t005

provide long-read sequencing of fragments ranging in size from 1000 to 20,000 bases or more. Such platforms would be more appropriate for barcodes such as rbcL

Genus or species misidentification is often attributed to missing plant sequences in reference databases [51]. Ecuador is one of the 20 megadiverse countries in the world, with two biodiversity hotspots [86]. Therefore, it is common to record new species in this tropical country, which may explain the under-representation of our sequences at the species level, in general databases. Plant taxonomy is important, especially in biodiversity hotspots, for several reasons: the contribution to knowledge by identifying and classifying species that are unknown or believed to be extinct, thus contributing to the advancement of knowledge and, consequently, to the establishment of conservation programmes for those species that require protection [87–89]. Taxonomic studies are also established as a baseline against which to work with programmes to monitor, track and make decisions on changes in species diversity due to habitat destruction and climate change. Furthermore, they facilitate the establishment of measures aimed at preventing the spread of invasive species that have the capacity to alter the integrity of ecosystems [90–92].

Although we demonstrated that the DNA methodology was superior for species-level identification to the SEM method, the latter allowed us to detect almost the same number of families within a sample [93]. The method of morphology and morphometric geometry, which uses high-quality 2D images (SEM) was applied a year before the current barcoding method to the same samples. We can attribute the low DNA quality of the samples in this study to the long period and conditions of storage, 3 years at 4 °C. In addition, the pollen samples did not undergo any prior washing or preservation

Table 6. Introduced flora identified as pollen sources for Ecuadorian stingless bees.

Plant	Origin	Vegetation type	Spanish common name
Coffea canephora (Rubiaceae)	W. Tropical Africa to S. Sudan and N. Angola	Shrub or tree up to 10 m	Café robusta
Brassica napus (Brassicaceae)	Europe to Mongolia and Pakistan, Canary Islands, N. Africa to Somalia and Arabian Peninsula	Weed	Canola, colza
Artocarpus sp. (Moraceae)	Tropical & Subtropical Asia to W. Pacific	Tree	Árbol de pan, frutipán
Choerospondias axillaris (Anacardaceae)	Nepal to S. China and Indo-China, Taiwan	Tree	NA
Solidago sp. (Asteraceae)	N. & Central America, Caribbean, Bolivia to Brazil and S. South America, Azores, Temp. Eurasia, NW. Africa.	Weed	Plumero Amarillo
Aster sp. (Asteraceae)	Eurasia to Jawa, NW. Africa, Subarctic America to NW. U.S.A.	Weed or small shrubs	NA
Dillenia sp. (Dilleniaceae)	W. Indian Ocean to SW. Pacific	Shrubs or trees up to 30 m	Falsa magnolia, manzana de elefante
Withania sp. (Solanaceae)	Tropical & S. Africa, Medit to Temp. Asia	Shrubs or weeds	Ginseng indio, hierba mora mayor
Secale cereale (Poaceae)	S. Türkiye	Weed	Centeno
Triticum turgidum (Poaceae)	E. Medit. To Iran and Xinjiang	Weed	Trigo
Ageratina adenophora (Asteraceae)	Mexico	Shrubs	Flor de la espuma
Laurus nobilis (Lauraceae)	Medit.	Shrubs or tree	Laurel, lauro

N: north. S: south. W: western. E: east. Medit: mediterranean. NA: not assigned

https://doi.org/10.1371/journal.pone.0323306.t006

method specific to DNA [94]. The ratio absorbance 260/280 of ≤ 1.6 may indicate the presence of proteins in samples [95]. The MiSeq recommendation is that a minimum of 50 ng to 500 ng of good-quality DNA should be utilized. It is imperative to note that DNA of substandard quality may contain traces of ethylenediaminetetraacetic acid (EDTA), organic contaminants such as ethanol, or other inhibitors that may interfere with library preparation (the case of this study) or DNA sequencing.

Twelve flora identified in this study are considered as introduced (non-native) in Ecuador [96]. Asia and Africa were the main origin sites which reflects the ecologically modified environment where the stingless bee sets are located. Forest trees were found as the main sources in a mixed native and exotic environment for Brazilian stingless bees [23] even when obtaining pollen and/or nectar, had higher energy costs than other shrubs and weeds. Stingless bees have been observed to follow a feeding pattern across all regions of their pantropical distribution. They visit both native plants and exotic species, including crops, ornamental plants, and weeds [19].

Plants in Melastomataceae, Myrtaceae, Asteraceae, Anacardiaceae, Euphorbiaceae, and Sapindaceae families are commonly reported to provide pollinic sources for stingless bees [97]. While reports of Polygonaceae, Solanaceae, Poaceae, Amaranthaceae, Dilleniaceae, and Araliaceae are frequent in other studies [98–100]. And as rarely reported we found Muntingiaceae and Nyctaginaceae families [22,101]. Some plants of Anacardiaceae family, genera *Croton* and *Cecropia,* and species such as *Trophis caucana, Secale cereale* include wind pollination (anemophilous) in their pollination [102–104]. The presence of the pollen in question in the nests of the stingless bees under study may be attributable to an indirect entry of pollen into the nests through wind currents or electromagnetic attraction to pollen charges that the bees carry in their corbiculae. It is recommended that the pollination mechanisms of these particular plants be studied to

ascertain whether their presence in the pollen pots was due to indirect contamination or whether it was attributable to the pollination activity of stingless bees.

Our results from Loja province, a tropical dry forest, indicated a greater diversity of pollen species during the rainy season. In the case of Orellana province, tropical rainforest, a greater diversity of pollen species was detected during the dry season [105]. Therefore we support the assertion that food stored is positively correlated with field food availability, which is greater when temperature and rainfall increase in the tropics [106]. Pollen richness and diversity inside the nests are positively related to environmental plant richness and the distance between nests and pollen sources [107].

In species of the genus *Melipona* sp., it has been observed that the flight distance may vary from 2 to 10 km when the resource reward is high, but it is surprising to observe this pattern in species such as *Scaptotrigona* sp., or *Tetragonisca* sp., for which there is no record of flight distances greater than one kilometer [97]. Thus, it is extremely important to maintain enough diverse floral sources around stingless bee nests, especially for those that are intended purely for the production of honey.

In Ecuadorian stingless bee keeping, we suggest differentiating the management of stingless bees according to the area in which the producer is located. Because when you generalize the bee keeping practices from one region to another without taking into consideration the months of local flowering, actions such as honey harvesting or nest division, can drastically affect the survival of the nests.

Conclusion

ITS2 region and rbcL gene increase the scope of taxonomical identification at the species level, by using them together. They also contribute to the understanding of the plant-pollinator relationship by revealing the origin and dispersal patterns of pollen as well as the specialisation of pollinators.

In Ecuador, tropical dry forests and tropical rainforests offer different pollen sources according to the season, an important factor to consider in the management of stingless bees, which must be differentiated for each region.

The pot-pollen richness included introduced flora, while the preferred vegetation type ranged from shrubs, weeds, and trees.

Understanding the available pollen sources is crucial for the effective management of stingless bees, identifying appropriate locations for meliponiculture, and cultivating or preserving plant species that are beneficial for honey-productive species in Ecuador.

Supporting information

S1 Table. Detailed list of Stingless bee species per sampled nest. The species were identified using two distinct methods: molecular biology and morphometric analysis. These methods were developed by two undergraduate students, Esteban Palacios and Ransey Pachacama in 2021 (unpublished information). ID code meaning: *H* meliponary or nest set, *N* nest, *P* pollen sample.
(DOCX)

Acknowledgments

The authors are grateful for the support of GIGA – Centre de Recherche Biomédicale Interdisciplinaire of the Université de Liège, especially to Mrs Latifa Karin from the Molecular and Computational Biology department during the sequencing process.

Author contributions

Conceptualization: Joseline Sofía Ocaña-Cabrera, Sarah Martin-Solano, Jorge Ron-Román, Claude Saegerman.

Data curation: Joseline Sofía Ocaña-Cabrera.

Formal analysis: Joseline Sofía Ocaña-Cabrera, Jose Rivas, Mutien-Marie Garigliany.

Funding acquisition: Sarah Martin-Solano, Jorge Ron-Román, Claude Saegerman.

Investigation: Joseline Sofía Ocaña-Cabrera.

Methodology: Joseline Sofía Ocaña-Cabrera, Sarah Martin-Solano, Claude Saegerman.

Project administration: Sarah Martin-Solano, Jorge Ron-Román, Claude Saegerman.

Resources: Sarah Martin-Solano, Claude Saegerman.

Software: Joseline Sofía Ocaña-Cabrera, Claude Saegerman.

Supervision: Sarah Martin-Solano, Mutien-Marie Garigliany, Claude Saegerman.

Validation: Joseline Sofía Ocaña-Cabrera, Claude Saegerman.

Visualization: Joseline Sofía Ocaña-Cabrera.

Writing – original draft: Joseline Sofía Ocaña-Cabrera.

Writing – review & editing: Sarah Martin-Solano, Jorge Ron-Román, Jose Rivas, Mutien-Marie Garigliany, Claude Saegerman.

References

1. Potts SG, Imperatriz-Fonseca V, Ngo HT, Aizen MA, Biesmeijer JC, Breeze TD, et al. Safeguarding pollinators and their values to human well-being. Nature. 2016;540(7632):220–9. https://doi.org/10.1038/nature20588 PMID: 27894123
2. Sabino W, Costa L, Andrade T, Teixeira J, Araújo G, Acosta AL, et al. Status and trends of pollination services in Amazon agroforestry systems. Agr Ecosyst Environ. 2022;335:108012.
3. Bawa KS. Plant-pollinator interactions in tropical rain forests. Annu Rev Ecol Evol Syst. 1990;21:399–422.
4. Borges RC, Brito RM, Imperatriz-Fonseca VL, Giannini TC. The value of crop production and pollination services in the eastern Amazon. Neotrop Entomol. 2020;49(4):545–56.
5. George TL, Zack S. Spatial and temporal considerations in restoring habitat for wildlife. Restor Ecol. 2001;9(3):272–9.
6. Anderson EK, Zerriffi H. Seeing the trees for the carbon: agroforestry for development and carbon mitigation. Climatic Change. 2012;115(3–4):741–57. https://doi.org/10.1007/s10584-012-0456-y
7. Brown AHD, Hodgkin T. Indicators of genetic diversity, genetic erosion, and genetic vulnerability for plant genetic resources. In: Ahuja MR, Jain SM, editors. Genetic Diversity and Erosion in Plants: Indicators and Prevention [Internet]. Cham: Springer International Publishing; 2015. p. 25–53. [cited 2025 Mar 25]. Available from: https://doi.org/10.1007/978-3-319-25637-5_2
8. Zattara EE, Aizen MA. Worldwide occurrence records suggest a global decline in bee species richness. One Earth. 2021;4(1):114–23. https://doi.org/10.1016/j.oneear.2020.12.005
9. Nath R, Singh H, Mukherjee S. Insect pollinators decline: an emerging concern of Anthropocene epoch. J Apic Res. 2023;62(1):23–38.
10. de Morais CR, Travençolo BAN, Carvalho SM, Beletti ME, Vieira Santos VS, Campos CF, et al. Ecotoxicological effects of the insecticide fipronil in Brazilian native stingless bees *Melipona scutellaris* (Apidae: Meliponini). Chemosphere. 2018;206:632–42.
11. Padilha AC, Piovesan B, Morais MC, de B. Pazini J, Zotti MJ, Botton M, et al. Toxicity of insecticides on neotropical stingless bees *Plebeia emerina* (Friese) and *Tetragonisca fiebrigi* (Schwarz) (Hymenoptera: Apidae: Meliponini). Ecotoxicology. 2020;29(1):119–28.
12. Piovesan B, Padilha AC, Morais MC, Botton M, Grützmacher AD, Zotti MJ. Effects of insecticides used in strawberries on stingless bees *Melipona quadrifasciata* and *Tetragonisca fiebrigi* (Hymenoptera: Apidae). Environ Sci Pollut Res Int. 2020;27(34):42472–80. https://doi.org/10.1007/s11356-020-10191-7 PMID: 32705562
13. Williams IH. The convention on biological diversity adopts the international pollinator initiative. Bee World. 2003;84(1):27–31.
14. Dar SA, Farook UB, Javeed K, Mir SH, Yaqoob M, Showkat A, et al. Pesticide legislation, national and international policies to maintain sustainable crop production through insect pollinator intervention. Int J Chem Stud. 2020;8(6):34–41.
15. Biesmeijer JC, Roberts SPM, Reemer M, Ohlemüller R, Edwards M, Peeters T, et al. Parallel declines in pollinators and insect-pollinated plants in Britain and the Netherlands. Science. 2006;313(5785):351–4. https://doi.org/10.1126/science.1127863 PMID: 16857940
16. Paz FS, Pinto CE, de Brito RM, Imperatriz-Fonseca VL, Giannini TC. Edible fruit plant species in the amazon forest rely mostly on bees and beetles as pollinators. J Econ Entomol. 2021;114(2):710–22. https://doi.org/10.1093/jee/toaa284 PMID: 33440000
17. Schilthuizen M, Kellermann V. Contemporary climate change and terrestrial invertebrates: evolutionary versus plastic changes. Evol Appl. 2014;7(1):56–67. https://doi.org/10.1111/eva.12116 PMID: 24454548

18. Everatt MJ, Convey P, Bale JS, Worland MR, Hayward SAL. Responses of invertebrates to temperature and water stress: a polar perspective. J Therm Biol. 2015;54:118–32.
19. Bueno FGB, Kendall L, Alves DA, Tamara ML, Heard T, Latty T, et al. Stingless bee floral visitation in the global tropics and subtropics. Glob Ecol Conserv. 2023;43:e02454.
20. Ollerton J, Winfree R, Tarrant S. How many flowering plants are pollinated by animals? Oikos. 2011;120(3):321–6. https://doi.org/10.1111/j.1600-0706.2010.18644.x
21. Abrahamczyk S, Kluge J, Gareca Y, Reichle S, Kessler M. The influence of climatic seasonality on the diversity of different tropical pollinator groups. PLoS One. 2011;6(11):e27115. https://doi.org/10.1371/journal.pone.0027115 PMID: 22073268
22. Gaona FP, Guerrero A, Gusmán E, Espinosa CI. Pollen resources used by two species of stingless bees (Meliponini) in a tropical dry forest of southern Ecuador. J Insect Sci. 2019;19(6):22. https://doi.org/10.1093/jisesa/iez125 PMID: 31853551
23. Martins AC, Proença CEB, Vasconcelos TNC, Aguiar AJC, Farinasso HC, de Lima ATF, et al. Contrasting patterns of foraging behavior in neotropical stingless bees using pollen and honey metabarcoding. Sci Rep. 2023;13(1):14474.
24. Slaa EJ, Sánchez Chaves LA, Malagodi-Braga KS, Hofstede FE. Stingless bees in applied pollination: practice and perspectives. Apidologie. 2006;37(2):293–315. https://doi.org/10.1051/apido:2006022
25. FAO, IZSLT, Apimondia, CAAS. Good beekeeping practices for sustainable apiculture [Internet]. Vol. 25. Roma: FAO Animal Production and Health Guidelines; 2021. [cited 2022 Sep 25]. Available from: http://www.fao.org/documents/card/en/c/cb5353en.
26. Roubik D, Aluja M. Flight ranges of *Melipona* and *Trigona* in tropical forest. J Kans Entomol Soc. 1983;56(2):217–22.
27. Rader R, Reilly J, Bartomeus I, Winfree R. Native bees buffer the negative impact of climate warming on honey bee pollination of watermelon crops. Glob Chang Biol. 2013;19(10):3103–10. https://doi.org/10.1111/gcb.12264 PMID: 23704044
28. Kellermann V, Overgaard J, Hoffmann AA, Fløjgaard C, Svenning JC, Loeschcke V. Upper thermal limits of *Drosophila* are linked to species distributions and strongly constrained phylogenetically. Proc Natl Acad Sci U S A. 2012;109(40):16228–33.
29. Li X, Ma W, Jiang Y. Honeybees (Hymenoptera: Apidae) adapt to the shock of high temperature and high humidity through changes in sugars and polyols and free amino acids. J Insect Sci. 2023;23(1):4. https://doi.org/10.1093/jisesa/iead002 PMID: 36695003
30. Ma C-S, Ma G, Pincebourde S. Survive a warming climate: insect responses to extreme high temperatures. Annu Rev Entomol. 2021;66:163–84. https://doi.org/10.1146/annurev-ento-041520-074454 PMID: 32870704
31. Vanbergen AJ. Initiative the IP. Threats to an ecosystem service: pressures on pollinators. Front Ecol Environ. 2013;11(5):251–9.
32. Giannini TC, Alves DA, Alves R, Cordeiro GD, Campbell AJ, Awade M, et al. Unveiling the contribution of bee pollinators to Brazilian crops with implications for bee management. Apidologie. 2020;51(3):406–21. https://doi.org/10.1007/s13592-019-00727-3
33. Ocaña-Cabrera JS, Liria J, Vizuete K, Cholota-Iza C, Espinoza-Zurita F, Saegerman C, et al. Pollen preferences of stingless bees in the Amazon region and southern highlands of Ecuador by scanning electron microscopy and morphometry. PLoS One. 2022;17(9):e0272580. https://doi.org/10.1371/journal.pone.0272580 PMID: 36126058
34. Silva MDE, Ramalho M, Monteiro D. Diversity and habitat use by stingless bees (Apidae) in the Brazilian Atlantic Forest. Apidologie. 2013;44(6):699–707. https://doi.org/10.1007/s13592-013-0218-5
35. Dell'Anna R, Cristofori A, Gottardini E, Monti F. A critical presentation of innovative techniques for automated pollen identification in aerobiological monitoring networks. In: Kaiser B, editor. Pollen, structure, types and effects. NOVA; 2010. p. 21.
36. Pospiech M, Javůrková Z, Hrabec P, Štarha P, Ljasovská S, Bednář J, et al. Identification of pollen taxa by different microscopy techniques. PLoS One. 2021;16(9):e0256808. https://doi.org/10.1371/journal.pone.0256808 PMID: 34469471
37. Pappas CS, Tarantilis PA, Harizanis PC, Polissiou MG. New method for pollen identification by FT-IR spectroscopy. Appl Spectrosc. 2003;57(1):23–7.
38. Zimmermann B. Characterization of pollen by vibrational spectroscopy. Appl Spectrosc. 2010;64(12):1364–73. https://doi.org/10.1366/000370210793561664 PMID: 21144154
39. Daood A, Ribeiro E, Bush M. Pollen grain recognition using deep learning. In: Bebis G, Boyle R, Parvin B, Koracin D, Porikli F, Skaff S, et al., editors. Advances in visual computing. Cham: Springer International Publishing; 2016. p. 321–30.
40. Peel N, Dicks LV, Clark MD, Heavens D, Percival-Alwyn L, Cooper C, et al. Semi-quantitative characterisation of mixed pollen samples using MinION sequencing and Reverse Metagenomics (RevMet). Methods Ecol Evol. 2019;10(10):1690–701. https://doi.org/10.1111/2041-210x.13265
41. Parducci L, Alsos IG, Unneberg P, Pedersen MW, Han L, Lammers Y, et al. Shotgun environmental DNA, Pollen, and Macrofossil analysis of late-glacial lake sediments from southern Sweden. Front Ecol Evol. 2019;7. https://doi.org/10.3389/fevo.2019.00189
42. Hebert PDN, Cywinska A, Ball SL, deWaard JR. Biological identifications through DNA barcodes. Proc Biol Sci. 2003;270(1512):313–21.
43. Ford C, Ayres K, Toomey N, Hider N, Van Alphen Stahl J, Kelly LJ, et al. Selection of candidate coding DNA barcoding regions for use on land plants. Bot J Linn. 2009;159(1):1–11.
44. Yao H, Song J, Liu C, Luo K, Han J, Li Y, et al. Use of ITS2 region as the universal DNA barcode for plants and animals. PLoS One. 2010;5(10):e13102. https://doi.org/10.1371/journal.pone.0013102 PMID: 20957043

45. Zhao L, Feng S, Tian J, Wei A, Yang T. Internal transcribed spacer 2 (ITS2) barcodes: a useful tool for identifying Chinese *Zanthoxylum*. Appl Plant Sci. 2018;6(6):e01157.

46. Zhang N, Zeng L, Shan H, Ma H. Highly conserved low-copy nuclear genes as effective markers for phylogenetic analyses in angiosperms. New Phytol. 2012;195(4):923–37. https://doi.org/10.1111/j.1469-8137.2012.04212.x PMID: 22783877

47. Zhang G-J, Dong R, Lan L-N, Li S-F, Gao W-J, Niu H-X. nuclear integrants of organellar DNA contribute to genome structure and evolution in plants. Int J Mol Sci. 2020;21(3):707. https://doi.org/10.3390/ijms21030707 PMID: 31973163

48. Kuzmin E, Taylor JS, Boone C. Retention of duplicated genes in evolution. Trends Genet. 2022;38(1):59–72. https://doi.org/10.1016/j.tig.2021.06.016 PMID: 34294428

49. Sullivan AR, Schiffthaler B, Thompson SL, Street NR, Wang XR. Interspecific plastome recombination reflects ancient reticulate evolution in *Picea* (Pinaceae). MBE. 2017;34(7):1689–701.

50. Loiseau O, Mota Machado T, Paris M, Koubínová D, Dexter KG, Versieux LM, et al. Genome skimming reveals widespread hybridization in a neotropical flowering plant radiation. Front Ecol Evol. 2021;9.

51. Newmaster SG, Fazekas AJ, Ragupathy S. DNA barcoding in land plants: evaluation of rbcL in a multigene tiered approach. Can J Bot. 2006;84(3):335–41.

52. Kress WJ, Erickson DL. A two-locus global DNA barcode for land plants: the coding rbcL gene complements the non-coding trnH-psbA spacer region. PLoS One. 2007;2(6):e508. https://doi.org/10.1371/journal.pone.0000508 PMID: 17551588

53. Nurhasanah S, Sundari , Papuangan N. Amplification and analysis of rbcL gene (Ribulose-1,5-Bisphosphate Carboxylase) of clove in Ternate Island. IOP Conf Ser: Earth Environ Sci. 2019;276(1):012061.

54. Wattoo JI, Saleem MZ, Shahzad MS, Arif A, Hameed A, Saleem MA. DNA barcoding: amplification and sequence analysis of rbcl and matK genome regions in three divergent plant species. Adv Life Sci. 2016;4(1):03–7.

55. Vasconcelos S, Nunes GL, Dias MC, Lorena J, Oliveira RRM, Lima TGL, et al. Unraveling the plant diversity of the Amazonian canga through DNA barcoding. Ecol Evol. 2021;11(19):13348–62. https://doi.org/10.1002/ece3.8057 PMID: 34646474

56. Dong W, Cheng T, Li C, Xu C, Long P, Chen C, et al. Discriminating plants using the DNA barcode rbcLb: an appraisal based on a large data set. Mol Ecol Resour. 2014;14(2):336–43. https://doi.org/10.1111/1755-0998.12185 PMID: 24119263

57. Maloukh L, Kumarappan A, Jarrar M, Salehi J, El-wakil H, Rajya Lakshmi TV. Discriminatory power of rbcL barcode locus for authentication of some of United Arab Emirates (UAE) native plants. 3 Biotech. 2017;7(2):144.

58. Chen S, Yao H, Han J, Liu C, Song J, Shi L, et al. Validation of the ITS2 region as a novel DNA barcode for identifying medicinal plant species. PLoS One. 2010;5(1):e8613. https://doi.org/10.1371/journal.pone.0008613 PMID: 20062805

59. Timpano EK, Scheible MKR, Meiklejohn KA. Optimization of the second internal transcribed spacer (ITS2) for characterizing land plants from soil. PLoS One. 2020;15(4):e0231436. https://doi.org/10.1371/journal.pone.0231436 PMID: 32298321

60. Claire-Iphanise M, Meyer RS, Taveras Y, Molina J. The nuclear internal transcribed spacer (ITS2) as a practical plant DNA barcode for herbal medicines. J Appl Res Med Aromat Plants. 2016;3(3):94–100.

61. Díaz M, Jarrín-V P, Simarro R, Castillejo P, Tenea GN, Molina CA. The Ecuadorian Microbiome Project: a plea to strengthen microbial genomic research. Neotrop Biodivers. 2021;7(1):223–37.

62. Mittermeier RA, Myers N, Hoffman M, Mittermeier C, Robles Gil P. Hotspots Revisited: Earth's Biologically Richest and Most Endangered Terrestrial Ecoregions. Mexico: CEMEX, S.A., Agrupación Sierra Madre, S.C.; 1999. p. 431.

63. Levin RA, Wagner WL, Hoch PC, Nepokroeff M, Pires JC, Zimmer EA, et al. Family-level relationships of Onagraceae based on chloroplast rbcL and ndhF data. Am J Bot. 2003;90(1):107–15. https://doi.org/10.3732/ajb.90.1.107 PMID: 21659085

64. Vere N de, Rich TCG, Ford CR, Trinder SA, Long C, Moore CW, et al. DNA barcoding the native flowering plants and conifers of wales. PLOS ONE. 2012;7(6):e37945.

65. Kolter A, Gemeinholzer B. Plant DNA barcoding necessitates marker-specific efforts to establish more comprehensive reference databases. Genome. 2021;64(3):265–98. https://doi.org/10.1139/gen-2019-0198 PMID: 32649839

66. Illumina. 16S metagenomic sequencing library preparation [Internet]. 2013. Available from: https://support.illumina.com/documents/documentation/chemistry_documentation/16s/16s-metagenomic-library-prep-guide-15044223-b.pdf

67. Dubois B, Debode F, Hautier L, Hulin J, Martin GS, Delvaux A, et al. A detailed workflow to develop QIIME2-formatted reference databases for taxonomic analysis of DNA metabarcoding data. BMC Genom Data. 2022;23(1):53. https://doi.org/10.1186/s12863-022-01067-5 PMID: 35804326

68. Moorhouse-Gann RJ, Dunn JC, de Vere N, Goder M, Cole N, Hipperson H, et al. New universal ITS2 primers for high-resolution herbivory analyses using DNA metabarcoding in both tropical and temperate zones. Sci Rep. 2018;8:8542.

69. CBOL Plant Working Group. A DNA barcode for land plants. Proc Natl Acad Sci U S A. 2009;106(31):12794–7.

70. Galimberti A, De Mattia F, Bruni I, Scaccabarozzi D, Sandionigi A, Barbuto M, et al. A DNA barcoding approach to characterize pollen collected by honeybees. PLoS One. 2014;9(10):e109363. https://doi.org/10.1371/journal.pone.0109363 PMID: 25296114

71. Richardson RT, Curtis HR, Matcham EG, Lin C-H, Suresh S, Sponsler DB, et al. Quantitative multi-locus metabarcoding and waggle dance interpretation reveal honey bee spring foraging patterns in Midwest agroecosystems. Mol Ecol. 2019;28(3):686–97. https://doi.org/10.1111/mec.14975 PMID: 30549365

72. Li H, Xiao W, Tong T, Li Y, Zhang M, Lin X, et al. The specific DNA barcodes based on chloroplast genes for species identification of Orchidaceae plants. Sci Rep. 2021;11(1):1424. https://doi.org/10.1038/s41598-021-81087-w PMID: 33446865
73. Gao T, Yao H, Song J, Zhu Y, Liu C, Chen S. Evaluating the feasibility of using candidate DNA barcodes in discriminating species of the large Asteraceae family. BMC Evol Biol. 2010;10:324. https://doi.org/10.1186/1471-2148-10-324 PMID: 20977734
74. Tahir A, Hussain F, Ahmed N, Ghorbani A, Jamil A. Assessing universality of DNA barcoding in geographically isolated selected desert medicinal species of Fabaceae and Poaceae. PeerJ. 2018;6:e4499.
75. Igbari A, Ogundipe O. Phylogenetic patterns in the tribe Acacieae (Caesalpinioideae: Fabaceae) based on rbcL, matK, trnL-F and ITS sequence data. Asia Pacific J Mol Biol Biotechnol. 2019;27(2):103–15.
76. Bell KL, Loeffler VM, Brosi BJ. An rbcL reference library to aid in the identification of plant species mixtures by DNA metabarcoding. Appl Plant Sci. 2017;5(3):apps.1600110. https://doi.org/10.3732/apps.1600110 PMID: 28337390
77. Xu S-Z, Li Z-Y, Jin X-H. DNA barcoding of invasive plants in China: a resource for identifying invasive plants. Mol Ecol Resour. 2018;18(1):128–36. https://doi.org/10.1111/1755-0998.12715 PMID: 28865184
78. Pang X, Song J, Zhu Y, Xu H, Huang L, Chen S. Applying plant DNA barcodes for Rosaceae species identification. Cladistics. 2011;27(2):165–70. https://doi.org/10.1111/j.1096-0031.2010.00328.x PMID: 34875771
79. Pere K, Mburu K, Muge EK, Wagacha JM, Nyaboga EN. Molecular discrimination and phylogenetic relationships of Physalis Species based on ITS2 and rbcL DNA barcode sequence. Crops. 2023;3(4):302–19. https://doi.org/10.3390/crops3040027
80. Nderitu KW, Ager E, Mecha E, Nyachieo A. DNA barcoding using its2 and RBCL markers for *Solanaceae* species identification. East Afr Med J. 2023;100(1):5567–74.
81. Ralte L, Singh YT. Use of rbcL and ITS2 for DNA barcoding and identification of *Solanaceae* plants in hilly state of Mizoram, India. Res Crops. 2021;22(3):616–23.
82. Wei L, Pacheco-Reyes FC, Villarreal-Quintanilla JÁ, Robledo-Torres V, Encina-Domínguez JA, Lara-Ramírez EE, et al. Effectiveness of DNA barcodes (rbcL, matK, ITS2) in identifying genera and species in Cactaceae. Pak J Bot [Internet]. 2024;56(5). [cited 2025 Mar 27]. Available from: https://www.pakbs.org/pjbot/paper_details.php?id=12089
83. Bell KL, Fowler J, Burgess KS, Dobbs EK, Gruenewald D, Lawley B, et al. Applying pollen DNA metabarcoding to the study of plant-pollinator interactions. Appl Plant Sci. 2017;5(6):apps.1600124. https://doi.org/10.3732/apps.1600124 PMID: 28690929
84. Prudnikow L, Pannicke B, Wünschiers R. A primer on pollen assignment by nanopore-based DNA sequencing. Front Ecol Evol. 2023; 11.
85. Cornman RS, Otto CRV, Iwanowicz D, Pettis JS. Taxonomic characterization of honey Bee (*Apis mellifera*) pollen foraging based on non-overlapping paired-end sequencing of nuclear ribosomal loci. PLoS One. 2015;10(12):e0145365. https://doi.org/10.1371/journal.pone.0145365 PMID: 26700168
86. Pullaiah T. Plant Biodiversity of Ecuador: A Neotropical Megadiverse country. In: Global Biodiversity: Vol 4. Selected Countries in the Americas and Australia: CRC Press; 2018. p. 590.
87. Al-Asif A, Nerurkar S. Taxonomy in crisis: addressing the shortage of taxonomists in a biodiversity hotspot era. JARS. 2024;1(2):1–4.
88. Sandall EL, Maureaud AA, Guralnick R, McGeoch MA, Sica YV, Rogan MS, et al. A globally integrated structure of taxonomy to support biodiversity science and conservation. TREE. 2023;38(12):1143–53.
89. Bevilacqua S, Anderson MJ, Ugland KI, Somerfield PJ, Terlizzi A. The use of taxonomic relationships among species in applied ecological research: baseline, steps forward and future challenges. Austral Ecol. 2021;46(6):950–64. https://doi.org/10.1111/aec.13061
90. Schouten MA, Barendregt A, Verweij PA, Kalkman VJ, Kleukers RMJC, Lenders HJR, et al. Defining hotspots of characteristic species for multiple taxonomic groups in the Netherlands. Biodivers Conserv. 2010;19(9):2517–36.
91. Marchese C. Biodiversity hotspots: a shortcut for a more complicated concept. Glob Ecol Conserv. 2015;3:297–309.
92. Raczkowski JM, Wenzel JW. Biodiversity studies and their foundation in taxonomic scholarship. BioScience. 2007;57(11):974–9.
93. Pornon A, Escaravage N, Burrus M, Holota H, Khimoun A, Mariette J, et al. Using metabarcoding to reveal and quantify plant-pollinator interactions. Sci Rep. 2016;6(1):27282.
94. Bell KL, de Vere N, Keller A, Richardson RT, Gous A, Burgess KS, et al. Pollen DNA barcoding: current applications and future prospects. Genome. 2016;59(9):629–40. https://doi.org/10.1139/gen-2015-0200 PMID: 27322652
95. Lucena-Aguilar G, Sánchez-López AM, Barberán-Aceituno C, Carrillo-Ávila JA, López-Guerrero JA, Aguilar-Quesada R. DNA Source selection for downstream applications based on DNA quality indicators analysis. Biopreserv Biobank. 2016;14(4):264–70. https://doi.org/10.1089/bio.2015.0064 PMID: 27158753
96. POWO. Plants of the World Online. Facilitated by the Royal Botanic Gardens, Kew [Internet]. 2023 [cited 2023 Nov 7]. Available from: http://www.plantsoftheworldonline.org/
97. Ramalho M. Stingless bees and mass flowering trees in the canopy of Atlantic Forest: a tight relationship. Acta Bot Bras. 2004;18(1):37–47. https://doi.org/10.1590/s0102-33062004000100005
98. Engel MS, Dingemans-Bakels F. Nectar and Pollen resources for stingless bees (Meliponinae, Hymenoptera) in Surinam (South America). Apidologie. 1980;11(4):341–50. https://doi.org/10.1051/apido:19800402

99. Saravia-Nava A, Niemeyer HM, Pinto CF. Pollen types used by the native stingless bee, Tetragonisca angustula (Latreille), in an Amazon-Chiquitano Transitional Forest of Bolivia. Neotrop Entomol. 2018;47(6):798–807. https://doi.org/10.1007/s13744-018-0612-9 PMID: 29949124

100. Absy ML, Rech AR, Ferreira MG. Pollen collected by stingless bees: a contribution to understanding Amazonian biodiversity. In: Vit P, Pedro SRM, Roubik DW, editors. Pot-Pollen in Stingless Bee Melittology. Cham: Springer International Publishing; 2018. p. 29–46.

101. Ghazi R, Zulqurnain NS, Azmi WA. Melittopalynological Studies of Stingless Bees from the East Coast of Peninsular Malaysia. In: Vit P, Pedro SRM, Roubik DW, editors. Pot-Pollen in Stingless Bee Melittology [Internet]. Cham: Springer International Publishing; 2018. p. 77–88. [cited 2021 Jul 26]. Available from: http://link.springer.com/10.1007/978-3-319-61839-5_6

102. Winiarczyk K, Tchórzewska D. Pollen grain on the compatible and incompatible stigma of *Secale cereale* L. Pobrane z czasopisma Annales C - Biología. 2013;68(2):45–55.

103. Bullock SH. Wind pollination of neotropical dioecious trees. Biotropica. 1994;26(2):172. https://doi.org/10.2307/2388806

104. Aguidelo Henao CA. Fenología de Especies Forestales de la Montaña del Ocaso, Quimbaya, Q. [Internet] [Undergraduated thesis]. [Colombia]: Universidad del Quindio; 2001 [cited 2025 Jan 13]. Available from: https://bdigital.uniquindio.edu.co/server/api/core/bitstreams/02db07d2-2e41-4b83-b5b1-ca9ca2dbe52b/content

105. Vaidya C, Fitch G, Martinez GHD, Oana AM, Vandermeer J. Management practices and seasonality affect stingless bee colony growth, foraging activity, and pollen diet in coffee agroecosystems. Agric Ecosyst Environ. 2023;353:108552.

106. Aleixo KP, Menezes C, Imperatriz Fonseca VL, da Silva CI. Seasonal availability of floral resources and ambient temperature shape stingless bee foraging behavior (*Scaptotrigona aff., depilis*). Apidologie. 2017;48(1):117–27.

107. Machado T, Viana BF, da Silva CI, Boscolo D. How landscape composition affects pollen collection by stingless bees? Landscape Ecol. 2020 Mar 1;35(3):747–59.
